# Postoperative Delirium in Elderly Patients Undergoing Hip Fracture Surgery in the Sugammadex Era: A Retrospective Study

**DOI:** 10.1155/2016/1054597

**Published:** 2016-02-22

**Authors:** Chung-Sik Oh, Ka Young Rhee, Tae-Gyoon Yoon, Nam-Sik Woo, Seung Wan Hong, Seong-Hyop Kim

**Affiliations:** ^1^Department of Anesthesiology and Pain Medicine, Konkuk University Medical Center, 120-1 Neungdong-ro, Gwangjin-gu, Seoul 05030, Republic of Korea; ^2^Institute of Biomedical Science and Technology, Konkuk University School of Medicine, Seoul, Republic of Korea

## Abstract

*Background.* Residual neuromuscular block (NMB) after general anesthesia has been associated with pulmonary dysfunction and hypoxia, which are both associated with postoperative delirium (POD). We evaluated the effects of sugammadex on POD in elderly patients who underwent hip fracture surgery.* Methods.* Medical records of 174 consecutive patients who underwent hip fracture surgery with general anesthesia were reviewed retrospectively to compare the perioperative incidence of POD, pulmonary complications, time to extubation, incidence of hypoxia, and laboratory findings between patients treated with sugammadex and those treated with a conventional cholinesterase inhibitor.* Results.* The incidence of POD was not significantly different between the two groups (33.3% versus 36.5%, resp.; *P* = 0.750). Postoperative pulmonary complications and laboratory findings did not showed significant intergroup difference. However, time to extubation (6 ± 3 versus 8 ± 3 min; *P* < 0.001) and the frequency of postoperative hypoxia were significantly lower (23% versus 43%; *P* = 0.010) in the sugammadex group than in the conventional cholinesterase inhibitor group.* Conclusion.* Sugammadex did not reduce POD or pulmonary complications compared to conventional cholinesterase inhibitors, despite reducing time to extubation and postoperative hypoxia in elderly patients who underwent hip fracture surgery under general anesthesia.

## 1. Introduction

Hip fracture surgery is performed primarily on elderly patients who are prone to various postoperative complications [1, 2], and delirium is the most common and dangerous psychiatric complication [3–5]. The anesthetic approach (regional versus general) to reduce postoperative delirium (POD) in this patient population remains controversial [4, 6]. As a history of anticoagulation medication use is often a contraindication to regional anesthesia, general anesthesia is frequently the only anesthetic option. Nondepolarizing neuromuscular blocking agents are not absolutely necessary for general anesthesia but they facilitate intubation and permit surgical levels of relaxation without the need for higher anesthetic concentrations that are often not tolerated by frail elderly patients. However, residual neuromuscular block (NMB) after emergence from general anesthesia has been associated with pulmonary dysfunction and hypoxia [7, 8]. To this end, sugammadex completely reverses NMB without cholinergic side effects [9] and reduces the frequency of postoperative pulmonary complications and hypoxia [10, 11] compared to conventional cholinesterase inhibitors.

Various precipitating factors have been reported for the development of POD, including older age, impaired cognitive status, other medical comorbidities, and various drugs [12]. Impaired postoperative pulmonary dysfunction and hypoxia have also been associated with POD in several studies [12, 13]. Considering that residual NMB leads to postoperative pulmonary complications and hypoxia [14, 15], improved pulmonary function with adequate oxygenation after reversing NMB with sugammadex could influence the reduction in postoperative cognitive dysfunction. However, the effects of sugammadex on POD have not been investigated.

We hypothesized that sugammadex would reduce the incidence of POD in elderly patients who had undergone hip fracture surgery under general anesthesia with a neuromuscular blocking agent through early recovery of pulmonary function and hypoxia compared to those who received a conventional cholinesterase inhibitor.

## 2. Materials and Methods

### 2.1. Study Population

This study was approved as a retrospective study by the Institutional Review Board of Konkuk University Medical Center, Seoul, South Korea (KUH1160075), and is registered at clinicaltrials.gov (NCT02305589). Written informed consent was not obtained from patients for the chart review component of the study. All data extracted from patient records were anonymized and deidentified prior to analysis as required for chart review studies by our Institutional Review Board. The medical records of patients who underwent hip fracture surgery under general anesthesia with a neuromuscular blocking agent at Konkuk University Medical Center from February 2012 to August 2014 were reviewed retrospectively. The patients were divided according to the use of sugammadex (S group) or conventional treatment (C group) to recover from NMB. A patient was excluded if any of the following criteria were met: (1) age < 60 years; (2) preoperative neurological or psychological problem including dementia, Parkinson's disease, and delirium; (3) preoperative abnormal findings on chest radiography; and (4) other concurrent surgeries.

### 2.2. Anesthetic Regimens

Anesthesia was induced and maintained according to the standard institutional regimen. Anesthesia was induced with propofol and maintained with sevoflurane under guidance of bispectral index (BIS) values maintained at 40–60. Remifentanil (5 ng/mL) was administered as a plasma target-controlled infusion using Minto's model [16] and was maintained during anesthesia. Rocuronium (0.6 mg/kg) was administered intravenously to induce muscle relaxation after loss of consciousness and was guided using peripheral neuromuscular transmission (NMT) monitoring (Datex-Ohmeda Division, GE Healthcare, Helsinki, Finland). Endotracheal intubation was performed when the train-of-four (TOF) count was zero. Additional rocuronium was administered to maintain TOF count at 1 to 2 under peripheral NMT monitoring during the operation. Sugammadex (2 mg/kg) was used in the S group for patients to recover from NMB, whereas neostigmine (0.05 mg/kg) and glycopyrrolate (0.01 mg/kg) were used for the C group. Above reversal agents were intravenously administered under peripheral NMT monitoring at the end of the operation. According to our institutional protocol, all extubation was performed after confirming a TOF ratio (T4/T1 ratio) of 0.9 and oxygen saturation (SpO_2_) > 95%, while the patient was breathing spontaneously with 1.0 of fraction of inspired oxygen (FiO_2_). Moreover, our institution obligated standard clinical muscle strength tests at postextubation period for patient safety, including the 5-second head lift test, swallowing test, and tongue depressor test, and inquiries as to the presence of diplopia were performed in the operating room to assure adequate recovery from NMB [17]. Delayed extubation was defined as the inability to proceed with extubation because of desaturation even while the patient was intubated or if neuromuscular recovery was incomplete 15 minutes after administration of sugammadex in the S group and after neostigmine was administered in the C group. The patients were transferred to the postanesthetic care unit (PACU) or intensive care unit (ICU) after extubation. An intravenous patient controlled analgesia (PCA) pump containing fentanyl (0.2 *μ*g/kg/hour, total dosage of fentanyl = 1500 *μ*g [30 mL] with 70 mL normal saline) was applied to all patients immediately before discharge from the operating room.

### 2.3. Postoperative Care

The decision for postoperative admission to the PACU or ICU was made by the attending anesthesiologist and surgeon following criteria in the institutional protocol. Patients were admitted to the PACU if they were hemodynamically stable, could respire spontaneously without difficulty, and were neurologically stable after extubation. Patients were admitted to the ICU if they required postoperative hemodynamic monitoring; ventilator support (respiratory failure if oxygen partial pressure [PaO_2_]/FiO_2_ ratio was < 200 mmHg after reversal of NMB; therapeutic ventilation for hypoxia or hypercarbia in a patient with a history of pulmonary disease); extensive neurological monitoring (Glasgow Coma Scale score < 8), were hemodynamically unstable during the operation due to massive bleeding, or had a clinical condition requiring ICU-level nursing care.

### 2.4. Assessment of Postoperative Delirium

If a patient developed delirium symptoms during the postoperative period, the surgeon checked their symptoms and consulted with a psychiatrist to assess and treat the delirium. Delirium symptoms were defined as the presence of any of the following: acute onset and fluctuating symptoms of inattention, disorganized speech, change in consciousness level, disorientation, impaired memory, perceptual disturbance, abnormal psychomotor activity, or altered sleep-wake cycle. The psychiatrist used the confusion assessment method (CAM) or the CAM-ICU rating for delirium [18, 19], as appropriate, and treated delirium using proper medication. When a patient was diagnosed or treated for POD in this way, the entire sequence was recorded in the medical chart. Therefore, in this study, the incidence of POD was assessed by reviewing medical records.

### 2.5. Postoperative Clinical Follow-Up

The following clinical data were obtained during the medical records review: intraoperative and postoperative volumes of transfused packed red blood cells, postoperative visual analogue scale score (0 [no pain] to 100 [worst pain imaginable]) at discharge from the PACU and on postoperative day 1, and delayed extubation. Postoperative chest radiography was performed in all patients and postoperative pulmonary complications (new onset abnormal findings on a postoperative chest radiograph with pulmonary symptoms, such as cough, sputum, and dyspnea) up to the first week postoperatively were evaluated. Postoperative hypoxia (SpO_2_ < 95%) up to the first 24 hours postoperatively, admission to the ICU, ICU stay duration, and hospital stay duration were also evaluated in all patients.

### 2.6. Perioperative Laboratory Findings

Hemoglobin, hematocrit, and serum levels of total protein, albumin, glucose, aspartate aminotransferase, alanine aminotransferase, creatinine, and high-sensitivity C-reactive protein were determined during the perioperative period by reviewing medical records.

### 2.7. Statistical Analysis

The incidence of POD in elderly patients was approximately 40% in a previous study [20]. A minimum difference in incidence of 50% was considered clinically significant. A sample size of 78 was determined to be required with a power of 0.8 and an *α*-value of 0.05. The groups were compared using the unpaired *t*-test, the chi-square test, or Fisher's exact test, as appropriate. Data were analyzed using SPSS ver. 18.0 software (SPSS Inc., Chicago, IL, USA). *P* < 0.05 was considered statistically significant.

## 3. Results

**Figure 1:**
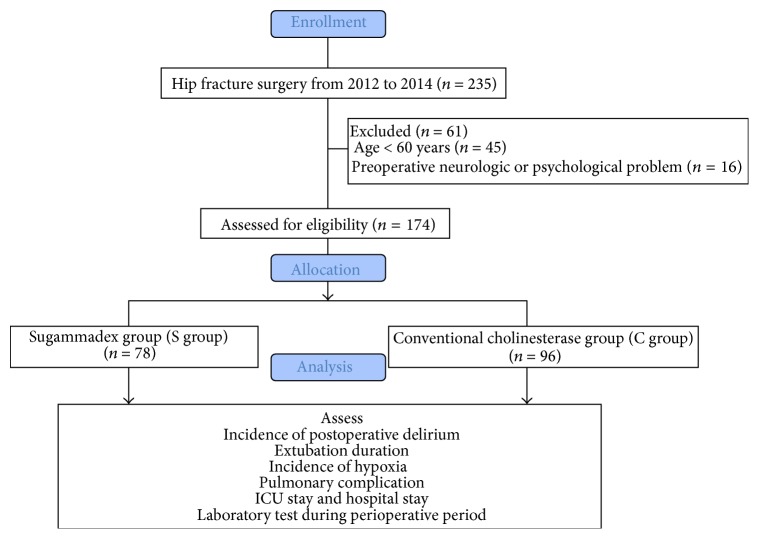
CONSORT flow diagram.

A total of 235 patients underwent hip fracture surgery under general anesthesia with a neuromuscular blocking agent from 2012 to 2014. The following number of patients met the exclusion criteria: 45 patients were < 60 years of age, and 16 patients had preoperative neurological or psychological problems, including dementia, Parkinson's disease, or delirium. Therefore, data from 174 patients who met the inclusion criteria were analyzed: 78 patients were treated with sugammadex and 96 patients were treated with a conventional cholinesterase inhibitor (Figure 1).

The overall incidence of POD was 35.1% (61/174) (S group, 33.3% [26/78], versus C group, 36.5% [35/96]; *P* = 0.750). The demographic characteristics were similar between the two groups (Table 1). Preoperative pulmonary function test did not show significant intergroup differences. The frequency of postoperative pulmonary dysfunction events, such as delayed extubation and pulmonary complications, was not different between the two groups (Table 2). However, the time to extubation was significantly shorter in the S group than in the C group (S group, 6 ± 3, versus C group, 8 ± 3 min; *P* < 0.001) and hypoxia up to postoperative 24 hours was significantly less often in the S group than in the C group (23% versus 43%; *P* = 0.010) (Table 2). No significant intergroup differences were detected in the number of ICU admissions, ICU or hospital stay duration, or laboratory findings. In all, 16 and 15 patients in the S and C groups, respectively, were admitted to the ICU (4 and 5 patients for delayed extubation; 2 and 2 for hemodynamic instability due to massive surgical bleeding; 8 and 5 for respiratory failure with PaO_2_/FiO_2_ ratio < 200 mmHg; and 2 and 3 for clinical conditions requiring ICU-level nursing care, resp.).

## 4. Discussion

Sugammadex did not reduce the incidence of POD compared to that of conventional cholinesterase inhibitors, although it was associated with a significantly shorter time to extubation and a lower incidence of postoperative hypoxia.

We hypothesized that sugammadex would reduce POD and decrease subsequent pulmonary complications after definite reversal of NMB relative to those of conventional cholinesterase inhibitors. However, sugammadex use could not reduce pulmonary complication in the present study. These results are in contradiction with the study of Ledowski et al. who found that postoperative residual block could increase postoperative pulmonary complication [10, 11]. The reason was assumed to be that all extubation procedure in the present study was achieved by confirming TOF ratio > 0.9 and there might be no difference of residual block in both groups. Even though sugammadex had benefit for shortening extubation time compared to conventional cholinesterase inhibitor, proper recovery from neuromuscular block could be also achieved by conventional cholinesterase inhibitor with meticulous TOF ratio monitoring in clinical aspect. The operation type and neuromuscular blocking agent in research of Ledowski et al. were not consistent in both groups and this could contribute to research bias. Several general operations including abdominal procedure could affect phrenic nerve function and could elicit diaphragm dysfunction related pulmonary complication, even without residual neuromuscular block [21]. Furthermore, inconsistent use of nondepolarizing neuromuscular blocking agent in research of Ledowski et al. could induce derangement to evaluating exact effect of sugammadex and neostigmine. On the other hand, the advantage of the present study was the consistent type of operation (only hip fracture surgery) and neuromuscular blocking agent (only rocuronium). It was known that postoperative pulmonary complication was lower after abdominal surgery than after orthopedic surgery [22], and proper comparison of postoperative pulmonary complication might be achieved in the present study.

Although sugammadex has benefit for lowering hypoxia up to 24 hours postoperatively in the present study, it did not extend to reducing POD. Oxidative stress resulting from pulmonary complications and hypoxia can induce a systemic inflammatory response that could result in neurospecific inflammation, and POD can be induced by the inflammatory response or hypoxia [23, 24]. The lack of a difference in the incidence of POD between the sugammadex and conventional cholinesterase inhibitors might have been associated with the relatively short half-life of sugammadex. The half-life of sugammadex is within 30 minutes [25], limiting its ability to prevent long-term postoperative pulmonary dysfunction at up to 1 week postoperatively [26], even if it is beneficial for early postoperative respiratory function. Therefore, sugammadex use was associated with a reduced frequency of postoperative hypoxia but was not associated with a significant reduction in the incidence of early POD in clinical aspect. Actually, POD was known as a complex process that is likely secondary to multiple factors [24]. Therefore, it was possible that influencing a single factor (postoperative hypoxia) could not reduce the incidence of this complication. Amorim et al. [27] reported a 40 min clinical benefit of sugammadex after NMB reversal. However, no significant clinical benefit of sugammadex, including cognitive function, was reported, which is consistent with its duration of action being too short to improve clinical outcomes immediately after surgery (15 minutes after NMB reversal) or on postoperative days 1 and 3. Therefore, although using sugammadex results in a fast and full recovery from NMB and a reduced incidence of hypoxia up to 24 hours postoperatively, its effects on POD appear to be limited.

Delirium is also associated with acetylcholine deficiency [28]. An anticholinergic agent such as glycopyrrolate is added when using conventional cholinesterase inhibitors to reduce muscarinic side effects [29]. In contrast, the main advantage of sugammadex is reversal of NMB without inhibiting acetylcholinesterase, which maintains better autonomic stability than when using conventional cholinesterase inhibitors with an anticholinergic agent. Therefore, we hypothesized that the absence of a cholinergic influence when using sugammadex would lower the incidence of POD, but this was not substantiated by our results. Cholinesterase inhibitors increase acetylcholine levels in the brain, and cholinesterase inhibitors, such as physostigmine or neostigmine, have been suggested for treating delirium [30–32]. Although the increase in acetylcholine levels caused by a cholinesterase inhibitor may prevent POD, these agents are accompanied by anticholinergics, such as atropine, which are known to induce delirium [33]. However, the anticholinergic glycopyrrolate used in our study has not been reported to induce delirium because it cannot cross the blood brain barrier [33, 34] and would not be expected to influence postoperative cognitive function. Consequently, we presume that using conventional cholinesterase inhibitors would not increase the incidence of POD, even when using an anticholinergic agent such as glycopyrrolate concomitantly. Similarly, sugammadex, despite not disturbing the cholinergic system, did not provide a benefit in prevention of POD compared to conventional cholinesterase inhibitors.

This study had several limitations. Firstly, even though we checked for residual NMB in the operating room during extubation, the actual TOF ratio at extubation moment could not be obtained through the medical record review, and the effect of conventional cholinesterase inhibitors could not be precisely compared to those of sugammadex. However, all extubation was performed after confirming the TOF ratio > 0.9 by our trained attending anesthesiologists as our institutional extubation protocol. In addition, all patients underwent one of the physical muscle strength tests such as the 5 s head lift test, swallowing test, or tongue depressor test. Even though there had been controversy, some previous studies demonstrated that these tests were correlated with the residual neuromuscular block  [17, 35]. Secondly, atropine was not used as an anticholinergic agent to reduce the muscarinic effect of conventional cholinesterase inhibitors as per our institutional protocol. Therefore, the effects of atropine on the central nervous system could not be evaluated and compared to those of sugammadex. However, time of emergence is significantly prolonged when atropine is used with neostigmine compared to when glycopyrrolate is used [36]. Therefore, mixing neostigmine and atropine appears not to be a good choice, and glycopyrrolate appears preferable as an anticholinergic agent in clinical practice. Finally, there are two forms of POD, hyperactive or hypoactive. In this study, the surgeon initially detected signs of POD symptoms and consulted a psychiatrist. As the psychiatrist did not screen for POD in all patients, we may have missed some cases of hypoactive delirium. This could be a study limitation according to the retrospective design. However, few studies have evaluated POD after sugammadex or conventional cholinesterase inhibitors were administered to patients. In spite of several limitations, this retrospective study can provide the basic relationship between decreased postoperative pulmonary functions after using different agents to reverse NMB and POD. Further well-designed prospective studies are required to obtain a clear association between these two factors.

In conclusion, use of sugammadex and conventional cholinesterase inhibitors resulted in a similar incidence of POD and pulmonary complications in elderly patients who had undergone hip fracture surgery with general anesthesia, although sugammadex reduced time to extubation and postoperative hypoxia for up to 24 hours postoperatively.

## Figures and Tables

**Table 1:** Patient demographic and clinical characteristics.

	S group (*n* = 78)	C group (*n* = 96)	*P*
Sex (M/F)	26/52	41/55	0.215
Age (years)	76 ± 7	75 ± 9	0.240
BMI (kg/m^2^)	22 ± 4	23 ± 3	0.738
Smoking Hx (pack-year)	3 ± 11	3 ± 10	0.903
Underlying disease			
Hypertension	56	59	0.198
DM	24	24	0.495
Dyslipidemia	21	23	0.727
Angina pectoris	9	13	0.820
Current medications			
Alpha-blocker	0	2	0.502
ARB	23	26	0.738
ACEi	2	2	1.000
Beta-blocker	15	13	0.407
CCB	30	30	0.340
Statin	12	23	0.186
LVEF (%)	67.5 ± 9.9	69.0 ± 6.3	0.334
PFT			
FEV1 (L)	1.7 ± 0.6	1.7 ± 0.5	0.598
FVC (L)	2.3 ± 0.9	2.5 ± 0.8	0.166
FEF 25–75% (L/s)	1.4 ± 0.7	1.3 ± 0.7	0.868
Operation			0.215
CR and IF	34	34	
OR and IF	7	14	
Bipolar	12	24	
THR	25	24	
Anesthesia time (min)	178 ± 46	186 ± 74	0.401
Operation time (min)	126 ± 48	122 ± 68	0.648
pRBC transfusion (unit)	1.4 ± 1.5	1.6 ± 2.4	0.510
Postop. bleeding (mL)	230 ± 292	298 ± 329	0.156
Postop. VAS1	39 ± 18	43 ± 14	0.124
Postop. VAS2	31 ± 11	32 ± 9	0.567

Data are numbers of patients or mean ± standard deviation.

S group, sugammadex group; C group, conventional cholinesterase inhibitor group; M, male; F, female; DM, diabetes mellitus; CVA, cerebrovascular accident; ARB, angiotensin receptor blocker; ACEi, angiotensin converting enzyme inhibitor; CCB, calcium channel blocker; NTG, nitroglycerin; LVEF, left ventricular ejection fraction before operation; PFT, pulmonary function test before operation; FEV1, forced expiratory volume in 1 second; FVC, forced vital capacity; FEF 25–75%, forced expiratory flow rate at 25–75%; Op, operation; CR and IF, closed reduction and internal fixation; bipolar, bipolar hemiarthroplasty; THR, total hip replacement; anesthesia time, time from arrival at operation room to discharge from the operating room; Op time, time from skin incision to dressing; pRBC, packed red blood cells; postop. bleeding, postoperative bleeding up to 24 hours postoperatively; postop. VAS1, postoperative visual analogue scale score at discharge from the postanesthesia care unit; postop. VAS2, postoperative visual analogue scale score on postoperative day 1.

**Table 2:** Clinical events and laboratory findings during the postoperative period.

	S group (*n* = 78)	C group (*n* = 96)	*P*
Time to extubation (min)	6 ± 3	8 ± 3	0.000
Delayed extubation	4	5	1.000
Pulmonary complications	16	19	1.000
Hypoxia	18	41	0.010
Admission to ICU	16	15	0.431
ICU stay duration (days)	0.7 ± 2.0	0.5 ± 2.2	0.481
Hospital stay duration (days)	17 (14–20)	16 (14–21)	0.816
Hematocrit (%)	29 ± 5	29 ± 5	0.272
Total protein (g/dL)	4.8 ± 1.1	4.8 ± 1.2	0.995
Albumin (g/dL)	2.5 ± 0.4	2.5 ± 0.8	0.567
Glucose (mg/dL)	140 ± 43	143 ± 56	0.708
AST (IU/L)	31 ± 15	33 ± 20	0.409
ALT (IU/L)	30 ± 51	20 ± 15	0.103
Creatinine (mg/dL)	0.7 ± 0.3	0.8 ± 0.5	0.121
HS-CRP (mg/dL)	3.4 ± 4.3	2.5 ± 3.3	0.150

Data are numbers of patients, mean ± standard deviation, or median (25%–75%).

S group, sugammadex group; C group, conventional cholinesterase inhibitor group; hypoxia, oxygen saturation <95% up to 24 hours postoperatively; ICU, intensive care unit; AST, aspartate aminotransferase; ALT, alanine aminotransferase; HS-CRP, high- sensitivity C-reactive protein.
